# Outcomes of conversion surgery for patients with locally advanced pancreatic cancer under strict indication criteria

**DOI:** 10.1007/s00595-025-03223-7

**Published:** 2026-02-02

**Authors:** Mihoko Yamada, Katsuhisa Ohgi, Ryo Ashida, Yoshiyasu Kato, Shimpei Otsuka, Hideyuki Dei, Katsuhiko Uesaka, Teiichi Sugiura

**Affiliations:** Division of Hepato-Biliary-Pancreatic Surgery, Shizuoka Cancer Center, 1007 Shimo-Nagakubo, Sunto-Nagaizumi, Shizuoka, 411-8777 Japan

**Keywords:** Pancreatic ductal adenocarcinoma, Conversion surgery, Locally advanced pancreatic cancer

## Abstract

**Purpose:**

Conversion surgery (CS) is performed for locally advanced pancreatic cancer (LAPC); however, the criteria for CS varies among institutions. This study aimed to evaluate the clinical outcomes of CS for LAPC based on strict indications at our center.

**Methods:**

This study included patients diagnosed with and treated for LAPC between 2011 and 2021. The CS criteria indicated that the tumors shrunk to the same status as resectable pancreatic cancer (RPC) on imaging and were maintained for > 3 months. Additionally, tumors that had shrunk to a size corresponding to borderline resectable pancreatic cancer (BRPC) were observed on CT.

**Results:**

Twenty-two patients underwent CS with a morbidity rate of 40.9% and no mortality. Overall survival after surgery in the LAPC group tended better than that in the BR and R groups (MST, LAPC, 59.7 months; BR, 38.6 months; R, 39.1 months; LAPC vs. BR, *p* = 0.085, vs. R, *p* = 0.065). Disease-free survival was similar across all resectability statuses: LAPC, 21.1 months; BR, 16.6 months; and R, 19.6 months (LAPC vs. BR, *p* = 1.000, vs. R, *p* = 1.000).

**Conclusion:**

CS in patients with LAPC under strict criteria showed promising outcomes, demonstrating the potential benefit of this approach in carefully selected patients.

**Supplementary Information:**

The online version contains supplementary material available at 10.1007/s00595-025-03223-7.

## Introduction

Pancreatic ductal adenocarcinoma (PDAC) has a poor prognosis, with a 5-year survival rate of 13% [1]. At the initial diagnosis, only 10–15% of patients are eligible for curative resection, underscoring the need for advanced therapeutic approaches [2]. Most patients with pancreatic cancer are diagnosed with unresectable disease according to resectability criteria, and multimodal therapy, such as chemotherapy or chemoradiotherapy, is the initial treatment [3, 4]. Recently, various systemic treatments have allowed conversion surgery (CS) to be performed in patients with a good response. These patients have better survival rates than non-resected patients [5–8].

CS has been widely performed since the introduction of FOLFIRINOX (oxaliplatin, irinotecan, 5-FU, and leucovorin) and GnP (nab-paclitaxel plus gemcitabine). However, there have been no randomized phase III trials of multimodal therapy, and several issues have been discussed. CS requires more advanced techniques than surgery for other resectability statuses [9]. Prolonged preoperative treatment affects histopathological results and recurrence patterns [10, 11]. The criteria for CS can vary among institutions, and a multidisciplinary conference is held to make the final decisions in areas of uncertainty [12]. This makes it difficult to compare the outcomes of each study. Many studies have evaluated the indications and outcomes of CS by stratifying patients with favorable prognostic factors or comparing outcomes with borderline resectable pancreatic cancer (BRPC) [6, 13]. Few reports have compared the outcomes of CS according to resectability status in patients with PDAC [14].

The outcome of CS depends on the resection criteria. In contrast, PDACs with other resectability statuses are clearly classified and resected based on certain standardized criteria [15]. This study aimed to clarify the clinical outcomes of CS for locally advanced pancreatic cancer (LAPC) based on strict indications at our center.

## Methods

### Patients

This study included 22 patients who were diagnosed with LAPC between 2011 and 2021, and subsequently underwent CS following neoadjuvant chemotherapy or chemoradiotherapy. Among them, 10 patients were diagnosed and underwent neoadjuvant therapy at our institution and 12 were referred from other hospitals for CS after neoadjuvant therapy. The determination of locally advanced disease was approved by a multidisciplinary conference, including surgeons, oncologists, radiologists, and endoscopists, at our hospital and confirmed that the cases referred from other hospitals for the purpose of CS were also unresectable retrospectively. All patients underwent computed tomography (CT) and were assessed according to the resectability criteria defined in the NCCN guidelines [16]. The short- and long-term outcomes of CS were compared with those of patients with RPC and BRPC who underwent surgery during the same period. To examine the differences in surgical outcomes and histopathological characteristics depending on the presence and duration of NAC, the RPC group included only patients who did not receive neoadjuvant treatment (NAT), whereas the BRPC group included only patients who received NAT. This study was approved by the Institutional Review Board of Shizuoka Cancer Center (J2023-71-2023-1-3).

### Neoadjuvant chemotherapy

Patients with LAPC were treated with modified FOLFIRINOX (mFFX) or gemcitabine plus nab-paclitaxel (GnP), and those with BRCP were treated with S-1 (40 mg/m^2^) with concurrent radiotherapy (50.4 Gy in 28 fractions), 4 cycles of mFFX, or 2 cycles of GnP. The GnP regimen consisted of gemcitabine (1000 mg/m^2^) and nab-paclitaxel (125 mg/m^2^) administered on days 1, 8, and 15, with each cycle repeated every four weeks. The mFFX regimen consisted of oxaliplatin (85mg/m^2^ for 2 h), leucovorin (200 mg/m^2^ for 2 h), and irinotecan (150mg/m^2^ for 90 min). This treatment was followed by a continuous infusion of fluorouracil (2400 mg/m^2^) for 46 h. The treatment cycles were repeated every two weeks [17].

### Criteria for conversion surgery at our institution

At our institution, CS for LAPC was performed based on strict criteria.

1. Tumor regression to RPC on imaging was maintained for more than three months.
2. Regression to BRPC on CT, with additional requirements of maintaining normal tumor marker levels (for at least six months) and showing no tracer activity on Fluorine-18 fluorodeoxyglucose positron emission tomography (FDG-PET) imaging.

CA19-9 was used as a tumor marker. Non-secretors of CA19-9 were considered as cases with no change. Normal and normalized tumor markers were defined as ≤ 37U/ml. Tracer activity was defined as the complete absence of abnormal FDG uptake in the primary tumor and metastatic sites, as visually assessed on FDG-PET by radiologists. Each case was approved by a multidisciplinary conference, including surgeons, oncologists, radiologists, and endoscopists, to ensure the appropriateness of CS.

These criteria were formally documented in 2019; however, similar principles were applied in earlier cases, with each case individually assessed and approved by a multidisciplinary conference.

### Evaluation at the diagnosis and before conversion surgery

Prior to initiating treatment, a series of examinations, including measurement of tumor markers, computed tomography (CT), ultrasonography, ethoxybenzyl-magnetic resonance imaging (EOB-MRI), FDG-PET, and endoscopic ultrasonography, were conducted to assess tumor extension. During the chemotherapy period, routine CT scans and laboratory tests, including those for tumor markers, were performed every 2 or 3 months. For patients demonstrating both tumor marker normalization and tumor shrinkage on CT, the tumor response was re-evaluated using RECIST version 1.1, and the response was classified as a complete response (CR), partial response (PR), stable disease (SD), or progressive disease (PD). The EOB-MRI and FDG-PET findings were re-assessed as part of this re-evaluation. Staging laparoscopy was performed when necessary.

### Conversion surgery

CS included pancreatoduodenectomy and distal pancreatectomy. When necessary, combined resection and reconstruction of the portal vein, superior mesenteric vein, and the hepatic artery were performed. The nerve plexus surrounding the superior mesenteric artery (SMA) was preserved with partial resection in cases of contact. Intraoperative pathological examination confirmed that the residual low-attenuation areas along the SMA, CHA, and CeA were free of cancer cells. If malignancy is detected in these regions, the resection is aborted. CS was also contraindicated in cases in which para-aortic lymph node metastasis or positive peritoneal cytology was confirmed intraoperatively. Postoperative complications were classified according to the Clavien-Dindo classification [18]. Postoperative pancreatic fistula was assessed using the International Study Group of Pancreatic Surgery (ISGPS) definition [19]. R0 resection was defined as the absence of microscopic tumor cells at the resection margins (0 mm rule). The treatment response was assessed using the Evans grading system [20].

### Postoperative treatment and follow-up

Adjuvant chemotherapy using either S-1 or gemcitabine was administered for six months when feasible [21]. During the first two years after resection, follow-up examinations, including physical examinations, laboratory tests, tumor marker assessments, and CT scans, were conducted every three months. If the patient remained recurrence-free for three years after resection, follow-up examinations were performed every six months. The median follow-up period for the censored patients was 37.5 months. Recurrence was defined based on radiological or histological evidence of tumor return following pancreatectomy and was categorized according to its location.

### Statistical analyses

Continuous variables are expressed as the median (range) and were compared using the Mann-Whitney U test. The chi-square test or Fisher’s exact test was performed for categorical variables, as appropriate. Overall survival (OS) was defined as the time interval between the date of diagnosis or surgery and death from any cause or last contact. Disease-free survival (DFS) was defined as the time interval between the date of the diagnosis or surgery and the date on which recurrence was confirmed. Survival curves were plotted using the Kaplan-Meier method and analyzed using the log-rank test. *p* < 0.05. All statistical analyses were performed using EZR (Saitama Medical Center, Jichi Medical University, Saitama, Japan) [22].

## Results

### Conversion rate and overall survival of patients with LAPC

**Fig. 1:**
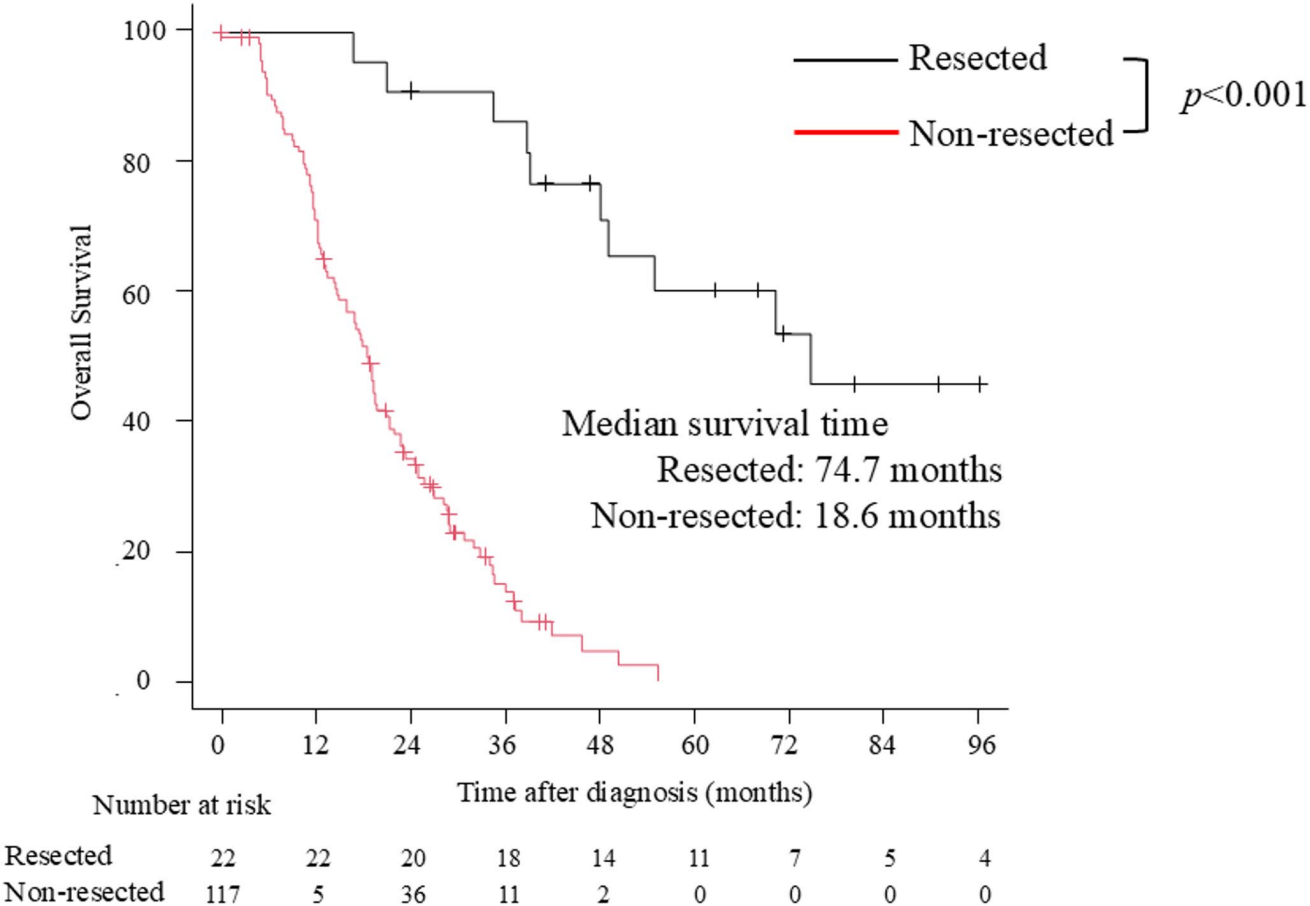
Kaplan-Meier survival curves for the overall survival of patients with LAPC who underwent resection (resected patients) and those who did not (non-resected patients). The 3- and 5-year OS rates were 86.1% and 60.2%, respectively, in resected patients and not available in 15.0%of non-resected patients (*p* < 0.001). The *p* value was obtained using a log-rank test to compare the two survival sets

A total of 127 patients with LAPC were treated at our hospital. Of these, 10 patients (7.9%) underwent CS. Including those referred by other hospitals after receiving NAT, 22 patients ultimately underwent CS and were classified as “resected patients.” Among the patients who underwent CS, 2 met Criterion 1 and 20 met Criterion 2, as previously described. In addition, 5 patients met the criteria for CS but ultimately did not undergo resection. Three cases were confirmed to be unresectable during laparotomy (2 with positive peritoneal cytology and 1 with perineural invasion around the right hepatic artery), and peritoneal dissemination was detected during staging laparoscopy in 1 of 2 the patients. One patient declined surgery despite the lack of apparent unresectable factors. The OS of the resected patients was significantly better than that of patients who did not undergo resection (non-resected patients). The 3-year OS and median survival time (MST) after the diagnosis in resected patients were 86.1% and 74.7 months, respectively, in comparison to 15.0% and 18.8 months in the non-resected patients (*p* < 0.001) (Fig. 1).

### Patient characteristics according to resectability status

Table 1 summarizes the clinical characteristics of patients based on their resectability status. The involvement of major arteries, specifically the SMA (*n* = 13), common hepatic artery (*n* = 3), and celiac artery (*n* = 5) was observed. The duration of NAT was significantly shorter in patients with BRPC (1.6 months) than in those with LAPC (10.8 months, *p* < 0.001). Additionally, the proportion of patients achieving a PR per the RECIST criteria was significantly higher in the LAPC group than in the BR group (90.9% vs. 17.5%, *p* = 0.017). In comparison to the BR and R groups, LAPC patients also exhibited smaller preoperative tumor sizes (LAPC vs. BR, *p* = 0.075; LAPC vs. R, *p* = 0.300) and significantly lower preoperative CA19-9 levels (LAPC vs. BR, *p* = 0.006; LAPC vs. R, *p* < 0.001). The median preoperative SUVmax on FDG-PET for LAPC patients was 2.4 (0-5.97), with 13 patients exhibiting a preoperative SUVmax of < 3.

**Table 1:** Background of patients according to resectability status

		LA (*n* =22)	BR (*n*=112)	*R* (*n*=363)	*p*	
					LA vs. BR	LA vs. *R*
Age*		68 (48–82)	67 (37–84)	72 (36–87)	1.000	0.019
Sex, male		14 (63.6%)	52 (46.8%)	199 (54.8%)	0.500	1.000
LA	SMA	13 (59.1%)	57 (50.9%)			
	CHA	3 (13.6%)	29 (25.9%)			
	CeA	5 (22.7%)	7 (6.2%)			
	rRHA		6 (5.4%)			
Diagnosis to operation (months)*		10.8 (4.2–18.9)	1.6 (0.4–7.1)		<0.001	
Preoperative therapy						
Chemotherapy		18 (81.8%)	76 (67.9%)		0.216	
GEM+nab-paclitaxel		10	62			
mFOLFIRINOX		5	10			
Others		3	4			
Chemoradiotherapy		4 (18.2%)	36 (32.1%)			
S-1+radiation		0	34			
Others		4	2			
Pre-treatment CA19-9 level (U/ml)*		50.8 (2–1178)	59.0 (2–4309)		0.707	
Pre-operative CA19-9 level (U/ml)*		15.0 (2–339)	30.5 (2–1650)	67.0 (2–16982)	0.006	<0.001
Pre-treatment tumor size (mm)*		31.5 (13–50)	27 (12–75)		0.207	
Pre-operative tumor size (mm)*		19.5 (10–30)	23.5 (7-65)	22.5 (1–63)	0.075	0.300
Pre-treatment FDG-PET, SUV max*		3.90 (0-6.50)				
Pre-operative FDG-PET, SUV max*		2.40 (0–5.97)				
RECIST	PR	20 (90.9%)	18 (17.5%)		0.017	
	SD	2 (9.1%)	82 (78.8%)			
	PD	0	4 (3.8%)			

LA, locally advanced pancreatic cancer; BR, borderline resectable pancreatic cancer; R, resectable pancreatic cancer; SMA, superior mesenteric artery; CHA, common hepatic artery; CeA, celiac artery; CA, carbohydrate antigen; FDG-PET, Fluorine-18 fluorodeoxyglucose positron emission tomography; PR, partial response; SD, stable disease. *, Median (range)

### Perioperative outcomes and pathological findings

The perioperative outcomes and histopathological findings according to the resectability status are shown in Table 2. The frequency of morbidity tended to be higher in the LAPC group (40.9%) than in the BR (25.9%, *p* = 0.590) and R (33.6%, *p* = 1.000) groups. There was one case of in-hospital mortality in a patient with RPC. R0 resection was achieved in 19 patients in the LAPC group (86.4%), which was comparable to the R0 resection rate in patients with BR (83.0%, *p* = 1.000) and lower than that in the R group (93.9%, *p* = 0.491).

**Table 2:** Perioperative outcomes according to resectability status

		LA (*n*=22)	BR (*n*=112)	*R* (*n*=363)	*p*	
					LA vs. BR	LA vs. *R*
Procedure	PD	15 (68.2%)	86 (76.8%)	244 (67.2%)	1.000	0.003
	DP	3 (13.6%)	16 (14.3%)	104 (28.7%)		
	DP-CAR	4 (18.2%)	8 (7.1%)	4 (1.1%)		
	TP	0	2 (1.8%)	11 (3.1%)		
Portal vein resection		11 (50.0%)	80 (71.4%)	109 (30.0%)	0.230	0.180
Hepatic artery resection		6 (27.3%)	17 (15.2%)	6 (1.7%)	0.214	<0.001
Operation time (min)**		464 (164–750)	439.5 (171–781)	400 (132–770)	0.723	0.036
Blood loss (ml)**		872 (160–2267)	747 (155–3927)	591 (0–3316)	0.360	0.031
Postoperative hospital stay (days)**		22 (10–71)	19 (8– 80)	20 (7–188)	0.700	1.000
Morbidity (CD≥3a)		9 (40.9%)	29 (25.9%)	122 (33.6%)	0.590	1.000
POPF(Grade≥B)		8 (36.4%)	24 (21.4%)	104 (28.7%)	0.510	1.000
Abdominal abscess		4 (18.2%)	7 (6.2%)	24 (6.6%)	0.250	0.200
DGE		1 (4.5%)	7 (6.2%)	38 (10.5%)	1.000	1.000
Abdominal bleeding		0	2 (1.8%)	5 (1.4%)	1.000	1.000
Margin status	R0	19 (86.4%)	93 (83.0%)	341 (93.9%)	1.000	0.491
Evans classification, ≥IIb		9 (40.9%)	28 (26.2%)		0.197	
Tumor diameter (mm)*		26 (5–70)	35 (15–75)	32 (10–100)	0.005	0.008
Differentiation	G2,3	14 (63.6%)	92 (82.1%)	246 (67.8%)	0.098	0.648
Lymph node metastasis		9 (40.9%)	74 (66.1%)	252 (69.4%)	0.032	0.009
Lymphatic invasion		13 (59.1%)	91 (81.2%)	301 (82.9%)	0.046	0.010
Venous invasion		10 (45.5%)	56 (50.0%)	225 (62.0%)	0.817	0.175
Perineural invasion		19 (86.4%)	105 (93.8%)	340 (93.7%)	0.640	0.540
Initiation of ACT		20 (90.9%)	92 (82.1%)	258 (71.1%)	0.528	0.049

LA, locally advanced pancreatic cancer; BR, borderline resectable pancreatic cancer; R, resectable pancreatic cancer; PD, pancreatoduodenectomy; DP, distal pancreatectomy; DP-CAR, DP with celiac axis resection; TP, total pancreatectomy; CD, Clavien-Dindo classification; POPF, postoperative pancreatic fistula; DGE, delayed gastric emptying; Mod, moderately differentiated adenocarcinoma; Por, poorly differentiated adenocarcinoma. *, Median (range); **, POPF was assessed by using the International Study Group of Pancreatic Surgery definition; ACT, Adjuvant chemotherapy

According to the histopathological findings, the response to NAT was evaluated as Evans grade ≥ IIb in 40.9% of the patients in the LAPC group, which was higher than that in the BR group (26.2%, *p* = 0.197). In comparison to the BR and R groups, the LAPC group had significantly smaller tumor sizes (LAPC vs. BR, *p* = 0.005; LAPC vs. R, *p* = 0.008). The rates of lymph node metastasis and lymphatic invasion in the LAPC group were lower than those in the BR (*p* = 0.032 and *p* = 0.046, respectively) and R groups (*p* = 0.026 and *p* = 0.029, respectively). The rate of adjuvant chemotherapy initiation in the LAPC group (90.9%) was higher than that in the BR (82.1%) and R (71.1%) groups.

### Overall and disease-free survival of the three groups

**Fig. 2:**
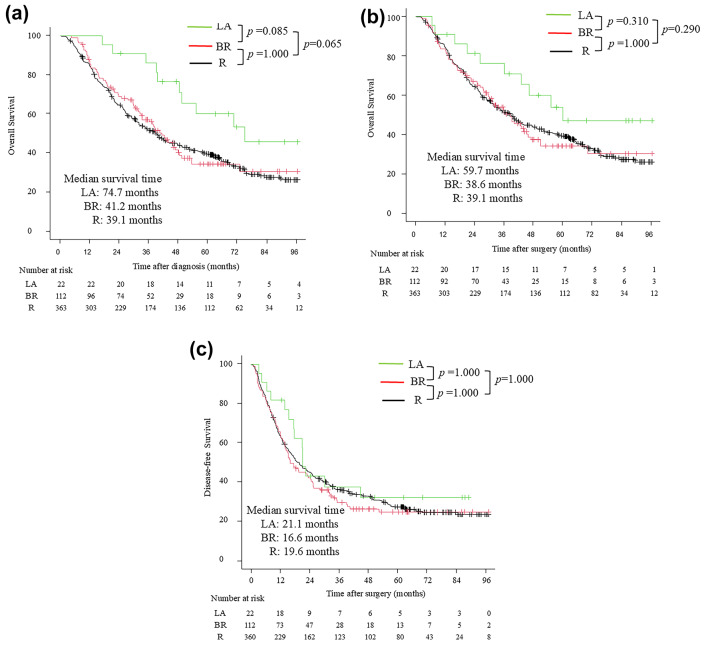
Kaplan-Meier overall and disease-free survival curves according to the resectability status. Overall survival (OS) was calculated from the time of the diagnosis (**A**) and the time of surgery (**B**), and disease-free survival (DFS) was calculated from the time of the surgery (**C**). *P* values were derived from log-rank tests. The 3- and 5-year OS in the LAPC, BR, and R groups were (**A**) 86.1% and 60.2%, 57.3% and 34.6%, and 51.6% and 39.8%, respectively, and (**B**) 71.2% and 47.7%, 50.7% and 34.6%, and 51.6% and 39.8%. The 3- and 5-year DFS in the LAPC, BR and R groups were (**C**) 37.9% and 32.5%, 30.2% and 25.2%, and 36.5% and 27.7%, respectively

OS after the diagnosis in the LAPC group tended better than that in the BR (MST, 74.7 months vs. 41.4 months, *p* = 0.085) and R (vs. 39.1 months, *p* = 0.065) groups (Fig. 2A). OS after surgery in the LAPC group was better than in patients with other resectability statuses (MST, LAPC, 59.7 months; BR, 38.6 months; R, 39.1 months; LAPC vs. BR, *p* = 0.310, LAPC vs. R, *p* = 0.290) (Fig. 2B). The median DFS after the surgery did not differ to a statistically significant extent among the 3 groups: LAPC, 21.1 months; BR, 16.6 months; R, 19.6 months (LAPC vs. BR, *p* = 1.000, vs. R, *p* = 1.000) (Fig. 2C).

### Recurrence

Table 3 shows the initial site of recurrence according to resectability status. In patients with LAPC, although multiple recurrence was the most frequent type of recurrence, the incidence of liver metastasis (7.1%) was lower than that in the BR (24.0%) and R (22.7%) groups.

**Table 3:** Initial recurrence site and according to resectability status

	LA (*n*=14)	BR (*n*=75)	*R* (*n*=238)	*p*	
				LA vs. BR	LA vs. *R*
Multiple	6 (42.9%)	20 (26.7%)	71 (29.8%)	1.000	1.000
Liver only	1 (7.1%)	18 (24.0%)	54 (22.7%)	0.860	0.940
Lung only	3 (21.4%)	6 (8.0%)	31 (13.0%)	0.440	1.000
Locoregional only	2 (14.3%)	16 (21.3%)	30 (12.6%)	1.000	1.000
Peritoneal only	1 (7.1%)	6 (8.0%)	16 (6.7%)	1.000	1.000
Pancreas only	0	2 (2.7%)	11 (4.6%)	1.000	1.000

LA, locally advanced pancreatic cancer; R, resectable pancreatic cancer; BR, borderline resectable pancreatic cancer

## Discussion

Although some studies on CS for LAPC have been reported over the past decades, the outcomes have not been fully compared according to resectability status. The outcomes of CS for LAPC have been compared with those of non-resected cases, BRPC cases, and, to a lesser extent, RPC cases. Patients with BRPC and RPC undergo resection according to standardized preoperative management, with less flexibility in treatment than those with LAPC. CS rates varied greatly from 16% to 86% and the reported MST ranged from 24.9 months to not reached [5–8], which makes it difficult to compare the results with these studies because the criteria for CS differed between institutions and also reflected the treatment status of the original LAPC population. A comparison of CS for LAPC by resectability status allowed us to confirm surgical outcomes, changes in pathological findings, and long-term outcomes according to the current criteria. In the present study, DFS after surgery was comparable to other resectability statuses, indicating that CS was performed appropriately in patients. In contrast, OS from the diagnosis and surgery tended to be better in patients with LAPC than in those with BR and R disease, suggesting that multimodal treatment, including resection, was appropriately administered.

Our criteria for CS were more rigorous than those reported previously [6, 13] and differed from those used at other institutions. At our institution, CS is only performed in highly selected patients. First, normalization and maintenance of CA19-9 levels for at least six months are required. The appropriate duration of NAT for CS has been reported 6–8 months based on the median progression-free survival of patients receiving GnP and FOLFIRINOX, 5.5 months and 6.8 months, respectively). In addition, the nadir CA19-9 level was reported to be observed at 4 months after the initiation of treatment [23]. Therefore, a 6-month observation period was applied after normalization of the CA19-9 levels. The definitive cutoff value of the CA19-9 level for CS has varied among studies, with several reports suggesting that normalization of CA19-9 could be considered a favorable prognostic factor, but not a criterion for resection [6, 13]. Recently, CA19-9 < 100 has been proposed as the normal level with a certain degree of consensus [24]. Maintaining tumor markers at normal levels or below a certain cutoff level is one way to ensure stable disease status. However, the duration for which CA19-9 is maintained, which is an appropriate indication for CS, is unclear. In the present study, most of our patients with CS normalized within three months, and most patients needed to be observed for an additional six months. Therefore, the median duration of preoperative treatment in patients was 10.8 months which was slightly longer than that in previous studies [6, 13]. Six months of observation was considered reasonable for determining tumor behavior.

Second, the FDG-PET performance was included as a criterion. FDG-PET has been reported to be effective for evaluating the efficacy of NAT for pancreatic cancer and its association with recurrence. CT findings after NAT sometimes cannot distinguish between viable tumors and fibrotic and desmoplastic changes [25]. Several studies have suggested that FDG-PET should be performed in addition to the measurement of tumor markers and CT to determine the indications for surgery after NAT [26, 27]. Although this study failed to show an association between the metabolic activity detected using FDG-PET and survival, FDG-PET played a supportive role in selecting patients for CS.

The features of histopathological findings after NAT, such as tumor size, lymph node metastasis, and microscopic findings, have been reported in previous studies [28]. In this study, lymph node metastasis and lymphatic invasion were significantly better in the LAPC group in comparison to the BR and R groups. In contrast, perineural invasion was not significantly different between the groups. Perineural invasion has been reported to play a driving role in PDAC progression from an early stage [29]. The rate of perineural invasion was reported to be lower in patients with NAT than in those without NAT [30]. One report found that the rate of perineural invasion did not differ markedly between patients with and without NAT (84.1% vs. 88.6%) in the ‘pre-FOLFIRINOX era’ [31]. In the present study, 20% of patients underwent NAT other than mFOLFIRINOX or GEM + nab-paclitaxel, which might have affected the outcomes. Another major concern in the pathological findings of patients with LAPC is the R0 resection rate. The R0 rate in patients with LAPC who underwent CS has been reported to be 75–91% [6, 13, 32–34]. The present study showed an R0 resection rate of 86.4%, which is comparable to the reported series. In comparison to other resectability statuses, the rate did not differ significantly, and the incidence of local recurrence alone was similar. All four patients with R1 experienced recurrence, including multiple recurrences in 3 patients and liver metastases in 1 patient. This suggests that a positive margin status is not simply a local finding but rather a reflex of tumor biology, especially in patients with LAPC.

The median OS and DFS after surgery in patients with LAPC have been reported to be 19.8–31.5 and 9.8–20.7 months, respectively [10, 13, 32, 33]. Few reports have shown better survival outcomes in patients with LAPC and BR disease in comparison to those with R disease. Our study showed that the survival outcome in patients with LAPC was better than that in those with BR and R disease. Especially after surgery, the DFS was comparable among the three groups, and the OS in patients with LAPC tended to be better than that in patients with BR or R disease. These trends were the same in the entire cohort, including all RPC and BRPC patients managed in the same period (Supplemental Fig. 1). Nine of 14 patients with LAPC who developed recurrence underwent chemotherapy and/or resection. These results showed that patients with a good performance status were fully treated at recurrence, indicating that CS can be safely performed and is a viable part of multidisciplinary treatment [35]. Furthermore, because patients with CS originally have a high response rate to chemotherapy or chemoradiotherapy, it can be assumed that they might also be more likely to respond to treatment after recurrence. Notably, patients in the CS group demonstrated better post-recurrence survival than those in other groups (Supplemental Fig. 2).

Several limitations of the present study warrant mention. First, it included a small number of patients who were managed at a single center; therefore, two possibilities should be considered: patients who could have benefited from resection may have dropped out due to strict resection criteria, and reassessment of NAT may have improved the outcomes of the patients in the R and BR groups. Regarding the former issue, some reports have expanded the indications, performed CS, and presented favorable results. Patients with SD or a minimal uptake of FDG may still have resectable tumors with acceptable outcomes, suggesting that our criteria might have been too strict. A comparison with a group showing a truly favorable response to treatment as a control group would require a prospective study or comparison between facilities with different indications. As for the latter issue, in particular, for this study period, the median duration of NAT in the BR group was 1.6 months, a relatively short period, due to the inclusion of all cases in which NAT was introduced. However, comparing CS patients who responded to NAT with those with other resectability statuses in the same period was useful in assessing the outcomes of CS. Second, although R0 resection was defined as the absence of invasive carcinoma within 1 mm in some reports [36], our study used a 0-mm rule. When comparing the three resectability statuses, the same rules were used to assess surgical margins. Third, the response evaluation of CA19-9 non-secretors is a limitation. These patients were classified as having “no change and alternative markers were not used. Although the DUPAN-2 is now routinely measured, it was not consistently assessed during the study period, which may have affected the accuracy of the response evaluation. Taken together, the outcomes of the comparison of the three different resectability statuses may provide a reference for deciding a strategy for CS.

## Conclusion

This study allowed us to observe the validity of CS by comparing surgical outcomes and pathological evaluations of our present CS policy among resectability statuses. CS was performed safely with favorable outcomes in patients who responded to NAT.

## Electronic Supplementary Material

Below is the link to the electronic supplementary material.

Supplementary Material 1: Supplemental Figure 1Kaplan-Meier overall and disease-free survival curves according to resectability status in the entire cohort. Overall survival (OS) was calculated from the time of diagnosis (A) and time of surgery (B), and disease-free survival (DFS) was calculated from the time of surgery (C). P values were derived from log-rank tests. The 3- and 5-year OS in the LAPC, BR, and R groups were (A) 86.1% and 60.2%, 52.9% and 31.9%, and 56.9% and 44.4%, respectively; and (B) 71.2% and 47.7%, 47.7% and 31.8%, and 57.0% and 44.4%. The 3- and 5-year DFS in the LAPC, BR, and R groups were (C) 37.9% and 32.5%, 27.9% and 23.4%, and 37.6% and 28.6%, respectively.Supplemental Figure 2Kaplan-Meier post-recurrence survival curves according to resectability status in the entire cohort.The 3- and 5-year after recurrence in the LAPC, BR, and R groups were 30.3% and 20.2%, 7.7% and 7.7%, and 12.7% and 5.1%. respectively.

## Abbreviations

PDAC: Pancreatic ductal adenocarcinoma
CA: Conversion surgery
LAPC: Locally advanced pancreatic cancer
BRPC: Borderline resectable pancreatic cancer
RPC: Resectable pancreatic cancer
SMA: Superior mesenteric artery
ISGPS: International Study Group of Pancreatic Surgery
OS: Overall survival
DFS: Disease-free survival
NAT: Neoadjuvant treatment
MST: Median survival time

## Declarations

### Animal studies

This study did not include material from other sources.
