# Supplementary material for: Outcomes of conversion surgery for patients with locally advanced pancreatic cancer under strict indication criteria
Source: Surg Today. 2026 Feb 2;56(7):1320–9. doi: 10.1007/s00595-025-03223-7 (PMC13303324; doi:10.1007/s00595-025-03223-7)

Supplemental Figure 1A

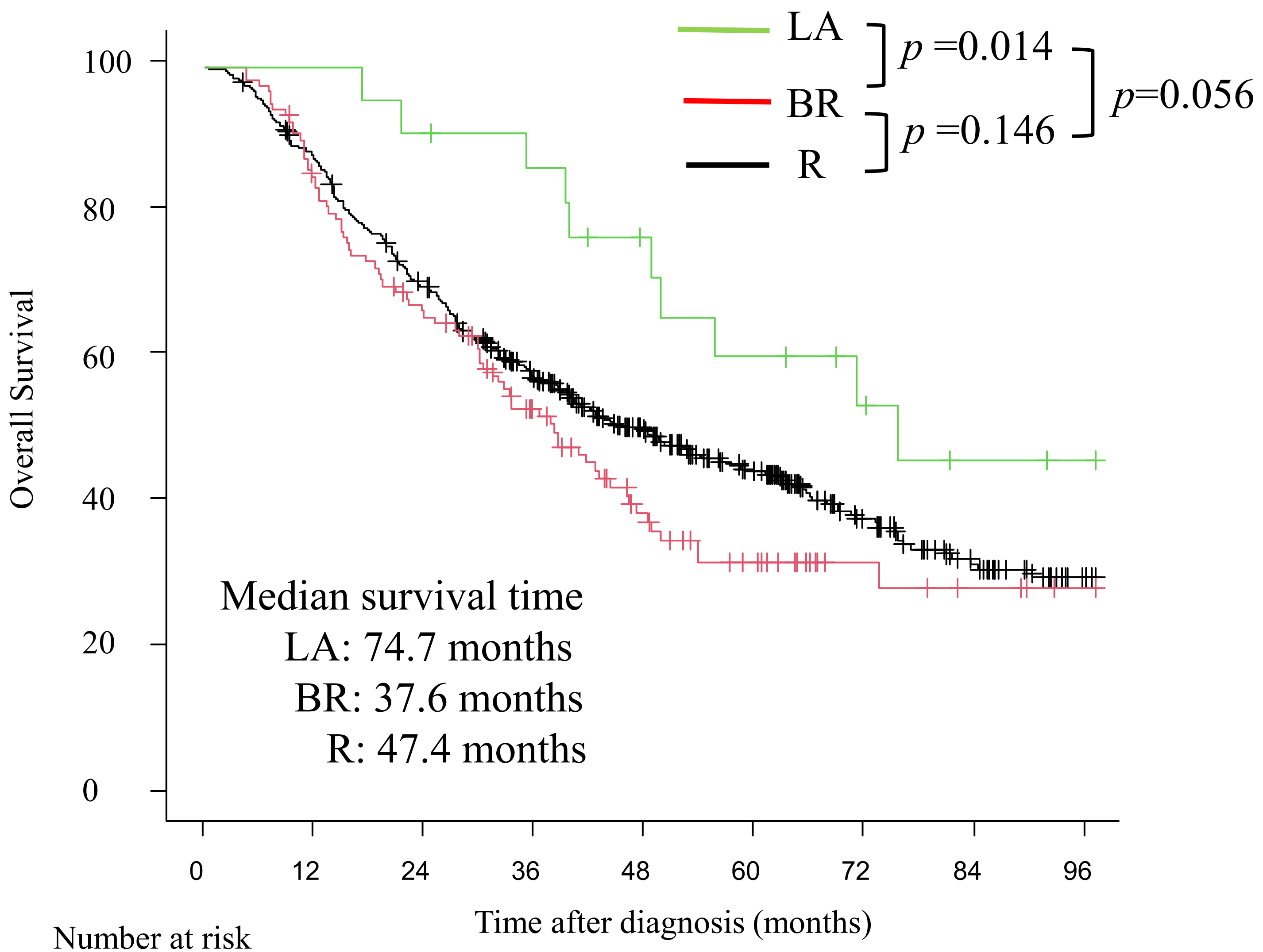

Number at risk

LA	22	22	20	18	14	11	7	5	4
BR	121	101	76	52	29	18	9	6	3
R	460	397	314	236	1167	122	69	39	13

Supplemental Figure 1B

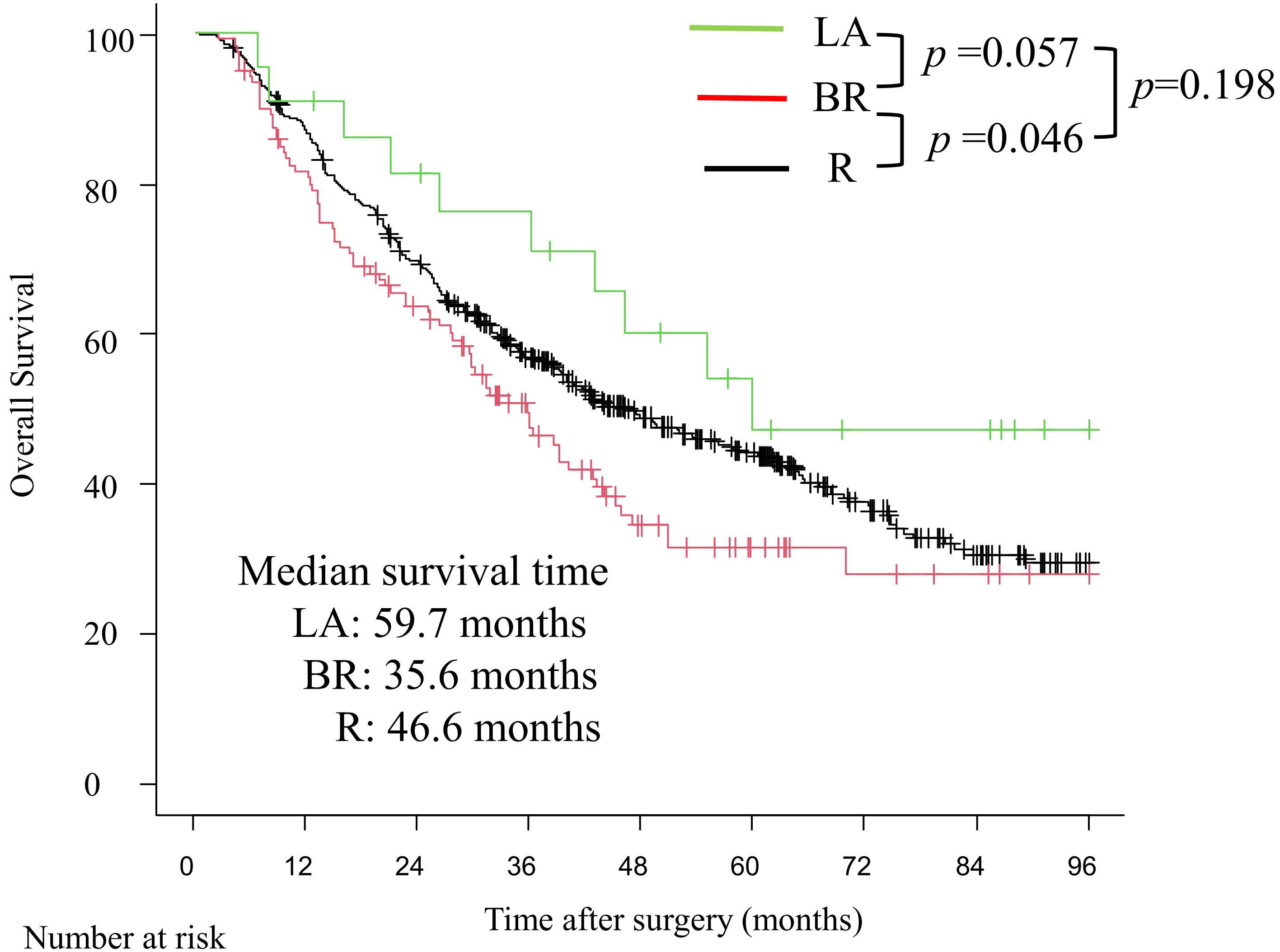

Number at risk

LA	22	20	17	15	11	7	5	5	1
BR	121	97	72	44	25	15	8	6	3
R	464	399	315	232	162	121	69	39	12

Supplemental Figure 1C

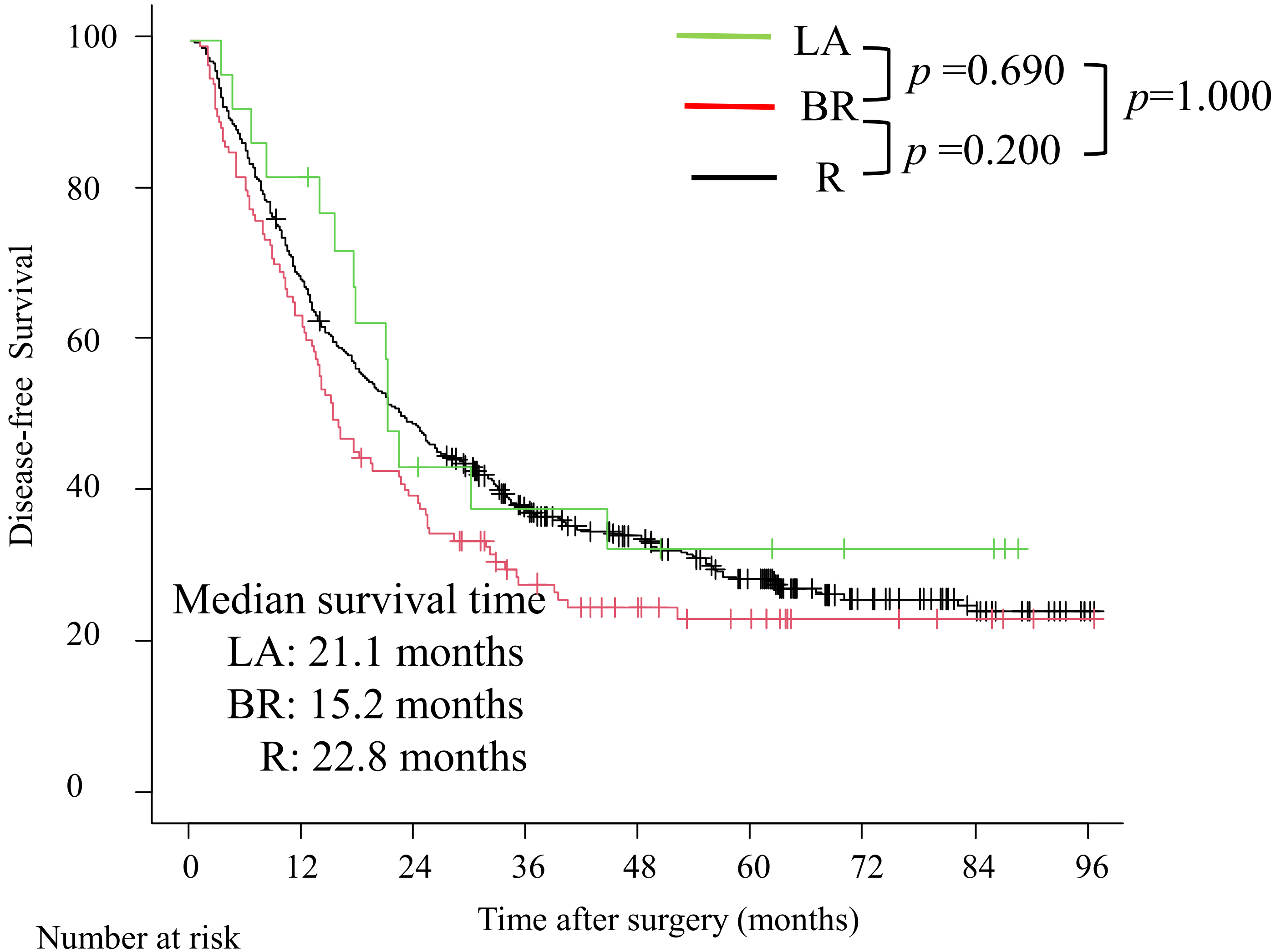

Number at risk

LA	22	18	9	7	6	5	3	3	0
BR	121	76	47	28	18	13	7	5	2
R	461	313	224	155	119	84	46	26	8

Supplemental Figure 2

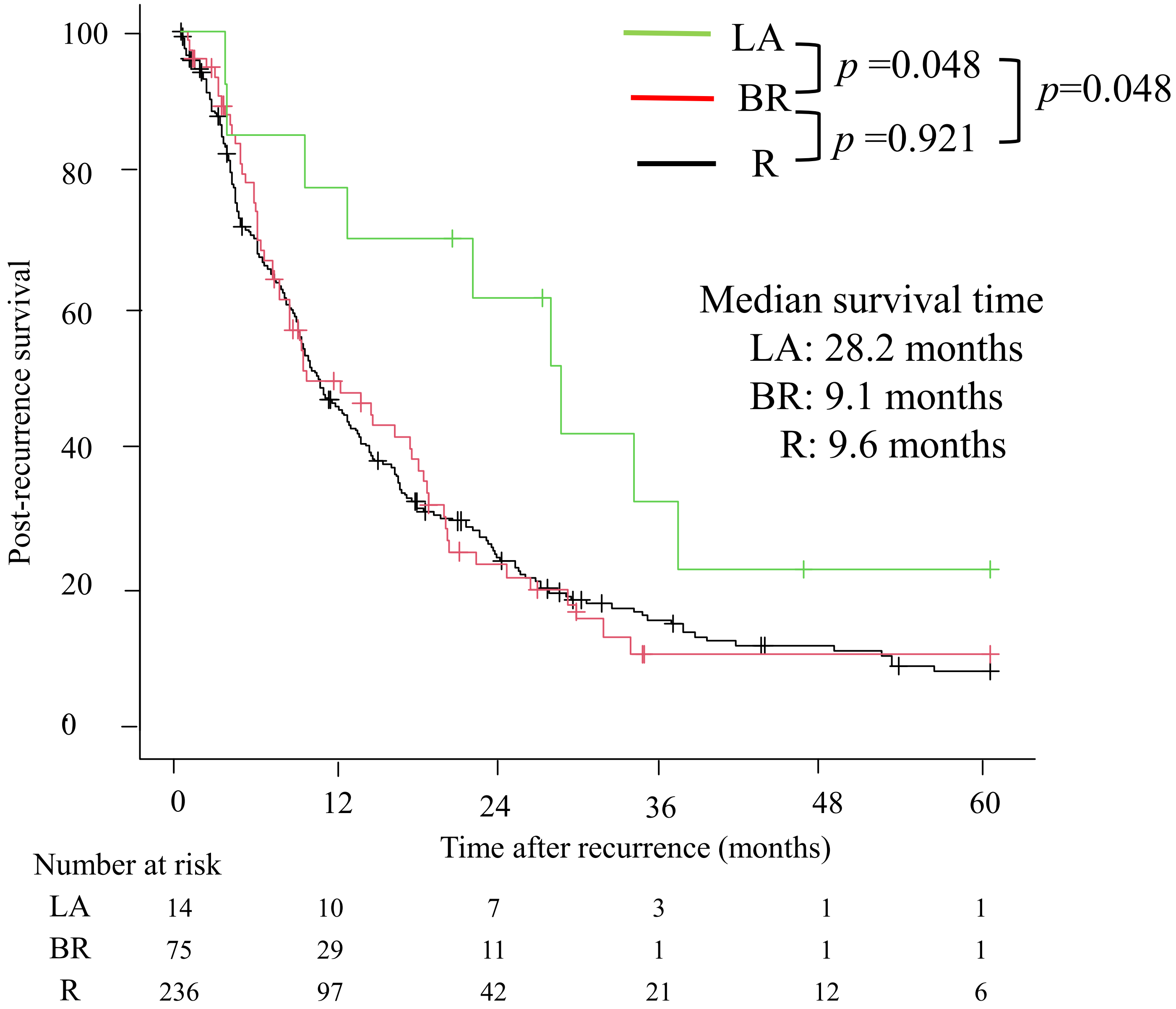

Supplement: Supplementary file 1 — Supplementary Material 1: Supplemental Figure 1Kaplan-Meier overall and disease-free survival curves according to resectability status in the entire cohort. Overall survival (OS) was calculated from the time of diagnosis (A) and time of surgery (B), and disease-free survival (DFS) was calculated from the time of surgery (C). P values were derived from log-rank tests. The 3- and 5-year OS in the LAPC, BR, and R groups were (A) 86.1% and 60.2%, 52.9% and 31.9%, and 56.9% and 44.4%, respectively; and (B) 71.2% and 47.7%, 47.7% and 31.8%, and 57.0% and 44.4%. The 3- and 5-year DFS in the LAPC, BR, and R groups were (C) 37.9% and 32.5%, 27.9% and 23.4%, and 37.6% and 28.6%, respectively.Supplemental Figure 2Kaplan-Meier post-recurrence survival curves according to resectability status in the entire cohort.The 3- and 5-year after recurrence in the LAPC, BR, and R groups were 30.3% and 20.2%, 7.7% and 7.7%, and 12.7% and 5.1%. respectively. [file 595_2025_3223_MOESM1_ESM.pdf]
